# Giant excitonic upconverted emission from two-dimensional semiconductor in doubly resonant plasmonic nanocavity

**DOI:** 10.1038/s41377-022-00860-2

**Published:** 2022-06-10

**Authors:** Pengfei Qi, Yuchen Dai, Yang Luo, Guangyi Tao, Liheng Zheng, Donglin Liu, Tianhao Zhang, Jiadong Zhou, Bo Shen, Feng Lin, Zheng Liu, Zheyu Fang

**Affiliations:** 1 School of Physics, State Key Laboratory for Mesoscopic Physics, Academy for Advanced Interdisciplinary Studies, Collaborative Innovation Center of Quantum Matter, Nano-optoelectronics Frontier Center of Ministry of Education, Peking University, Beijing, 100871 China; 2 Institute of Modern Optics, Nankai University, Tianjin Key Laboratory of Micro-scale Optical Information Science and Technology, Tianjin, 300350 China; 3 Photonics Research Center, School of Physics, MOE Key Lab of Weak-Light Nonlinear Photonics, and Tianjin Key Lab of Photonics Materials and Technology for Information Science, Nankai University, Tianjin, 300071 China; 4 School of Electrical and Electronic Engineering, Nanyang Technological University, Singapore, 639798 Singapore

**Keywords:** Nanophotonics and plasmonics, Nanocavities

## Abstract

Phonon-assisted upconverted emission is the heart of energy harvesting, bioimaging, optical cryptography, and optical refrigeration. It has been demonstrated that emerging two-dimensional (2D) semiconductors can provide an excellent platform for efficient phonon-assisted upconversion due to the enhanced optical transition strength and phonon-exciton interaction of 2D excitons. However, there is little research on the further enhancement of excitonic upconverted emission in 2D semiconductors. Here, we report the enhanced multiphoton upconverted emission of 2D excitons in doubly resonant plasmonic nanocavities. Owing to the enhanced light collection, enhanced excitation rate, and quantum efficiency enhancement arising from the Purcell effect, an upconverted emission amplification of >1000-fold and a decrease of 2~3 orders of magnitude in the saturated excitation power are achieved. These findings pave the way for the development of excitonic upconversion lasing, nanoscopic thermometry, and sensing, revealing the possibility of optical refrigeration in future 2D electronic or excitonic devices.

## Abstract (web summary)

Based on locally enhanced electromagnetic field and accelerated spontaneous emission rate, excitonic upconversion enhancement of more than 1000-fold was achieved in plasmonic nanocavity coupled transition metal dichalcogenide monolayers.

## Graphical abstract

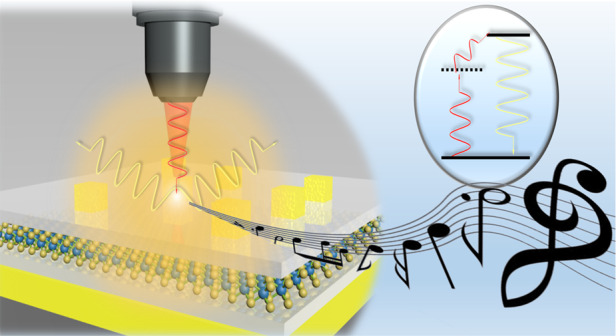

## Introduction

Photon upconversion is an anti-Stokes process for emitting a photon at an energy higher than excitation photon energy through a variety of mechanisms, ranging from high harmonic generation, multiphoton absorption, and Auger recombination to phonon scattering^1–7^. Relevant to this work, phonon-assisted anti-Stokes emission has been demonstrated to be an appealing possibility for fundamental studies and applications, including bioimaging and phototherapy^8–15^, volumetric displays^16^, upconversion lasers^17–19^, optical writing^20^, optical tweezers^21^, optical cryocooling^22^, nanoscale thermometry, and sensing^6^. Accordingly, efficient upconversion photoemission has been extensively investigated in various luminescent systems such as organic dyes, quantum dots, nanobelts, carbon nanotubes, and especially lanthanide-doped upconversion nanoparticles^23–26^.

The efficiency of phonon-assisted upconversion can be enhanced by emitters with a high optical transition strength or by realizing resonant conditions-that is, the incident or/and emitted photon energy matches the resonance level of the material system. Owing to the reduced dielectric screening and enhanced Coulomb attraction, the electron-hole pairs formed in monolayer semiconductors have a Bohr radius of ∼1 nm and binding energy of 500 meV (over an order of magnitude greater than that of conventional semiconductors). Thus, the fundamental optoelectronic properties are determined by excitonic effects at both cryogenic and room temperatures^27–34^. The optical transition strength and phonon-exciton interaction effects are strongly enhanced in comparison to traditional 3D and quasi-2D semiconductors^33,35^. Hence, monolayer semiconductors can provide a great platform for fundamental studies and applications of efficient phonon-assisted upconversion^35,36^, such as excitonic upconversion lasing and optical refrigeration of excitonic devices. Although the upconversion from 2D semiconductors has been observed^35,36^, the intensity/efficiency of the upconversion intensity is weak, and research on the further enhancement of upconverted emission at low-threshold excitation intensity for 2D excitons is still in an early stage.

Coupling the quantum emitters to an optical cavity can significantly change the interaction between the emitter and its local optical environment^37,38^. Moreover, the resonance frequencies of localized surface plasmons (LSPs) can be conveniently tailored by changing the size, shape, and interparticle separation. Here, we report the enhanced upconverted emission of two-dimensional excitons in doubly resonant plasmonic nanocavities. Through the integration of monolayer WSe_2_ into designed plasmonic nanocavities that doubly resonate with the incident or/and emitted photons, the deep subwavelength mode volume of cavity resonances can provide locally enhanced electromagnetic fields and thereby enhance the phonon-mediated optical absorption. In addition, the spontaneous emission rate of the emitter can be accelerated via the Purcell factor in the weak coupling regime, leading to plasmon-enhanced luminescence^39–42^. Eventually, up to 1100-fold enhancement of the upconversion photoluminescence (PL) in monolayer WSe_2_ is achieved, and the saturated excitation energy is reduced by 2~3 orders of magnitude, which decreases energy consumption. This work offers new opportunities in upconversion devices and applications, including infrared detectors, laser refrigerators, biochemical sensors, and energy harvesters.

## Results

### Design and characterization of plasmonic upconverter devices

**Fig. 1:**
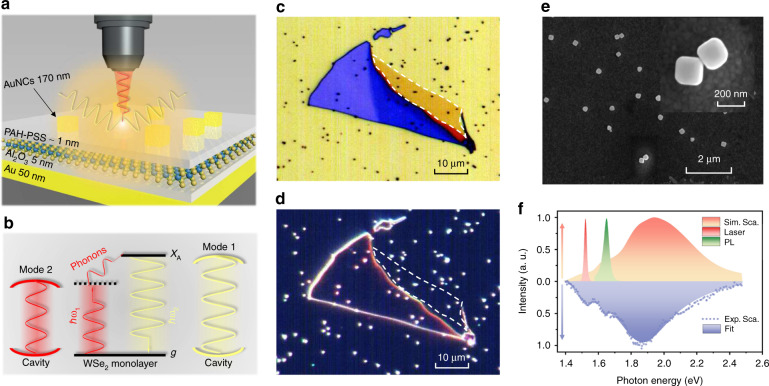
Design and characterization of plasmonic upconverter devices. **a** Schematic of the designed Au nanocube/WSe_2_/substrate plasmonic upconverter devices. **b** Desired band alignment diagram of monolayer WSe_2_ in plasmonic nanocavities and the photon upconversion process of 2D excitons. **c** Bright- and **d** dark-field microscope optical images of a representative sample. **e** Scanning electron micrograph of 170 nm Au nanocubes on the substrate, and the inset shows a magnified image from the top view. **f** Excitation laser and monolayer WSe_2_ PL spectra overlapping the simulated scattering spectrum of a plasmonic nanocavity, which is consistent with experimental results. The excitation laser and upconverted emission spectra are doubly resonant with plasmonic cavity modes at 1.52 and 1.67 eV, respectively. All the spectra are normalized

To enhance excitonic upconverted emission in doubly resonant plasmonic nanocavities, the nanoparticle-on-mirror geometry (equivalent to gap-mode patch antennas) was used, placing mechanically exfoliated monolayer WSe_2_ in the gaps between nanoparticles and a mirror underneath^38,42^. Figure 1a illustrates a schematic of the designed Au nanocube (AuNC)/WSe_2_/substrate plasmonic upconverter devices, where the substrate consists of a 5 nm Al_2_O_3_ spacer and a 50 nm Au layer evaporated on a Si/SiO_2_ wafer. The desired band alignment diagram of monolayer WSe_2_ embedded in plasmonic nanocavities is depicted in Fig. 1b, where the photon upconversion process of 2D excitons is also sketched. In monolayer WSe_2_, the electrons in the ground state are excited and relaxed as excitons by simultaneously absorbing a photon and phonons (red arrow), where the photon energy ℏω_1_ is located at the long-wavelength tail of the absorption spectrum. Then, the formed excitons can recombine via spontaneous emission of upconverted photons with energy ℏω_2_ > ℏω_1_ (yellow arrow). For the elaborate plasmonic nanocavities, the two cavity modes are doubly resonant with the incident and emitted photon energies to guarantee that both the excitation and emission processes are enhanced. At lower energy gain and temperature, a series of excitonic states, including spin-triplet trions (T_T_) and spin-singlet trions (T_S_), can be upconverted to neutral excitons under resonant conditions, as well as upconverting via dark excitons to bright intravalley excitons^43^. The superposition of excitonic state resonances and plasmonic nanocavity resonances is expected to achieve even more appealing upconversion enhancements.

Figure 1c, d shows bright- and dark-field optical micrographs of a representative sample, respectively. The exfoliated monolayer WSe_2_ was transferred onto a 5 nm Al_2_O_3_/50 nm Au/SiO_2_/Si substrate fabricated by evaporation coating. Then, AuNCs were sparsely deposited onto the monolayer WSe_2_ flake by drop casting (see Supplementary Fig. S1 for more details). Monolayer WSe_2_ and AuNCs were separated with an organic adhesive layer [poly(allylamine) hydrochloride (PAH)/poly(sodium-p-styrenesulfonate) (PSS)] with a thickness of ∼1 nm (see Supplementary Fig. S2 for more details). The AuNCs were synthesized using the seed-mediated method, and an average side length of ~170 nm was obtained according to the SEM image depicted in Fig. 1e. The spacer layers among the Au film, WSe_2_, and AuNCs can prevent hot carriers that decay from LSPs or surface plasmon polariton (SPP) from being injected into monolayer WSe_2_.

Figure 1f presents the simulated and experimental scattering spectra to confirm the optimal matching among the excitation laser, upconversion photons, and cavity modes. Noticeably, the simulated spectrum is consistent with the experimental result. The spectra of the excitation laser and monolayer WSe_2_ PL overlap with the simulated scattering spectrum of a plasmonic nanocavity. This means that the excitation laser (1.52 eV) and upconverted emission spectra (peak at 1.65 eV) are doubly resonant with plasmonic cavity modes at 1.52 and 1.67 eV, respectively. The prominent peak of the upconverted emission spectrum can be attributed to the neutral exciton (X) in the monolayer WSe_2_. Clearly, the emitted photon energy is 130 meV higher than that of the incident photons, which is four times greater than the results in ref. ^35^ The photon energy difference ΔE is much larger than the intrinsic phonon energy of ~30 meV in the monolayer WSe_2_ (see Supplementary Fig. S3 for more details), which implies that multiple phonons are involved in the excitonic upconverted emission. Note that phonon-assisted absorption and emission have been extensively studied in past decades^44^, and the rigorous theory of multiphonon-assisted absorption and emission in semiconductors was developed in ref. ^45^.

### Phonon-assisted excitonic upconverted emission of monolayer WSe_2_

**Fig. 2:**
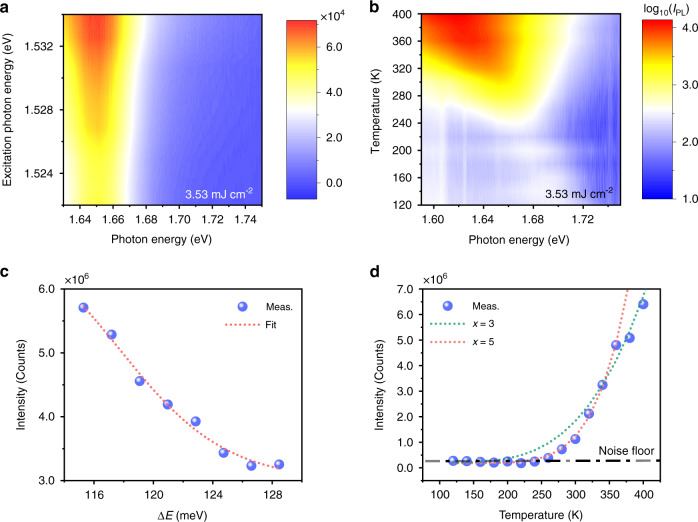
Phonon-assisted excitonic upconverted emission of monolayer WSe_2_. **a** Excitation photon energy-dependent upconverted emission spectra. **b** Excitation photon energy-dependent integrated upconverted emission intensity extracted from (**a**). The red dotted line represents the fitted result based on the Boltzmann function. **c** Temperature-dependent upconverted emission spectra for excitation photon energy at 1.52 eV. **d** Temperature-dependent integrated upconverted emission intensity extracted from (**c**). The upconverted emission intensity is proportional to $\bar n^x$, where $\bar n = 1/\left[ {\exp \left( {\hbar \omega _q/k_BT} \right) - 1} \right]$ is the average population of phonon gas in monolayer WSe_2_ and *x* is the involved phonon number in single unconverted emission. The green and red dotted lines show the fitted results for *x* = 3 and 5, respectively

As depicted in Fig. 1b, the excitonic upconverted emission is determined by incident photons and the phonons in monolayer WSe_2_. Figure 2a shows the incident photon energy-dependent upconverted emission spectrum, where the excitation photon energy ranging from 1.522 to 1.534 eV was precisely controlled by a wavelength-tuneable femtosecond pulse oscillator. As the excitation photon energy gradually overlaps with the exciton emission peak, the spectral shape and peak position of upconverted emission are constant, whereas the intensity evidently increases. To quantify the dependence of the total upconverted emission intensity on the excitation photon energy, the integrated intensity of the upconversion spectra (Fig. 2a) as a function of the energy difference ΔE between the excitonic emissions and the excitation photons is presented in Fig. 2b. The experimental results can be fitted well by the excitons obeyed classical Boltzmann function.

As the quanta of the crystal vibrational field, phonon energy, and density are determined by the lattice temperature. In addition, the energy gap-related excitonic emission peak of semiconductors depends on temperature, considering the temperature-dependent lattice dilatation and electron-lattice interaction^46^. Therefore, temperature plays a critical role in the spectral shape, intensity, and peak position of excitonic upconverted emission. Figure 2c presents the temperature-dependent excitonic upconverted emission spectra (see Supplementary Fig. S14 for more details). It can be clearly observed that the exciton peak broadens and red-shifts with increasing temperature, which is consistent with the exciton PL spectra under standard excitation. The broadening and red-shifting of spectra can be explained well by the well-known Varshni equation and the interaction of excitons with the longitudinal-acoustical and longitudinal-optical phonon modes of the lattice (see Supplementary Fig. S4 for more details). Figure 2d depicts the temperature-dependent upconverted emission intensity. As the temperature decreases, the energy difference ΔE between the excitonic emissions and the exciting photons increases gradually due to the blue-shift of the exciton peak, and the density decrease. Thus, the total upconverted emission intensity drops dramatically, so the detectable upconverted emission (above the noise floor) cannot be found for temperatures lower than 250 K. Considering that multiple phonons are involved in the upconversion process, the experimental results in Fig. 2b are proportional to $\bar n^x$, where $\bar n = 1/\left[ {\exp \left( {\hbar \omega _q/k_BT} \right) - 1} \right]$ is the temperature-dependent average population of phonon gas in monolayer WSe_2_ and *x* is the involved phonon number in single upconversion emission. The green and red dotted lines show the fitted results for *x* = 3 and 5, respectively. This illustrates that the higher the temperature is, the fewer phonons are needed in single upconverted emission, which is consistent with the decrease in ΔE with increasing temperature (Fig. 2c). In conclusion, the excitation photon energy- and temperature-dependent measurements show behavior consistent with the physical scenario depicted in Fig. 1b, thereby providing undoubtable evidence for phonon-assisted excitonic upconverted emission. It is worth mentioning that the electron concentration in the monolayer WSe_2_ influences the content of different exciton species, which is reflected in the upconversion^36^, especially when the excitation light resonates with these low energy excitonic species. Although beyond the scope of this work, achieving upconversion enhancement under different electron concentrations remains an exciting topic to study since various methods including ambient atmosphere^47^, photogating effect^48^, and hBN encapsulation^43^ can be applied to modulate electron concentrations of TMDs in addition to electrical nodes.

### Enhanced excitonic upconverted emission in plasmonic cavity

**Fig. 3:**
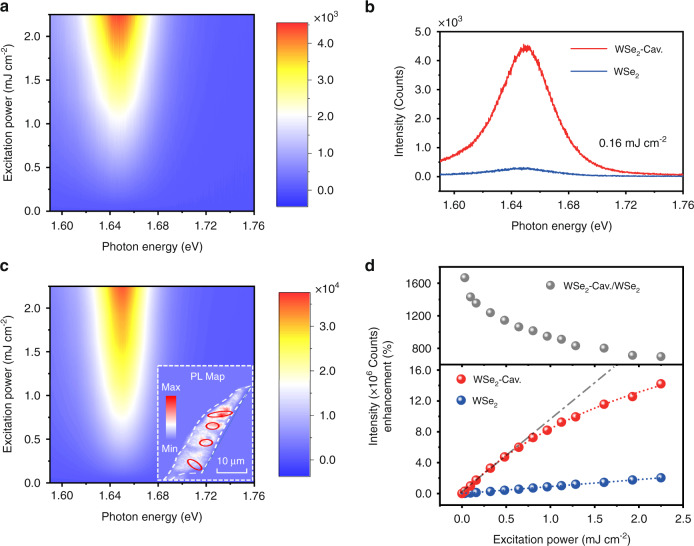
Upconversion amplified by the plasmonic cavity. **a** Excitation power-dependent upconverted emission spectra for monolayer WSe_2_ on SiO_2_/Si. **b** Enhanced upconverted emission spectra of monolayer WSe_2_ in the designed plasmonic cavity at an excitation power of 0.16 mJ/cm^2^. **c** Excitation power-dependent upconverted emission spectra for monolayer WSe_2_ in the designed plasmonic cavity. Inset: PL map of the plasmonic nanocavity effect on exciton emission, where the nanocubes and monolayer WSe_2_ are marked by a red solid line and white dashed line. **d** Excitation power-dependent integrated upconverted emission intensity (down) and enhancement (top) for monolayer WSe_2_ in the designed plasmonic cavity. The experimental results (scattering points) can be fitted well by the blue and red dotted lines based on Eq. (2)

To explore the enhancement of excitonic upconverted emission in the plasmonic cavity, we performed excitation power-dependent measurements with an excitation photon energy of 1.52 eV at room temperature and ambient conditions. First, experiments were carried out on monolayer WSe_2_ transferred to SiO_2_/Si. Figure 3a presents the excitation power-dependent upconverted emission map. Here the energy fluence of a single pulse on a unit area was adopted to exhibit the change of excitation power. As the excitation power increases, the intensity evidently increases, whereas the spectral shape and peak position of upconverted emission are permanent, illustrating that the thermal and renormalization effects can be reasonably neglected at such an excitation power level. In addition, the dependence of the total upconverted emission intensity on the excitation power is shown in Fig. 3d, which exhibits an obvious linear relation. The possibility of nonlinear optical generation through the observed PL upconversion, such as two-photon excitation-induced emission^49,50^ and exciton Auger scattering^51,52^ can be ruled out; that is, the phonon-assisted excitonic upconverted emission is further confirmed.

In well-designed plasmonic upconverter devices (Fig. 1a, f), the excitation laser and upconverted emission spectra are doubly resonant with plasmonic cavity modes. Enhanced upconverted emission can be expected compared with monolayer WSe_2_ on SiO_2_/Si under the same conditions. Figure 3b shows the upconverted emission spectra of WSe_2_ on SiO_2_/Si and the plasmonic cavity at an excitation power of 0.16 mJ/cm^2^, where a 13.5-fold enhancement is obtained (see Supplementary Fig. S15 for more samples). To explicitly reveal the dependence of the enhancement on the excitation power, the excitation power-dependent upconverted emission map for WSe_2_ in the plasmonic cavity was measured and is plotted in Fig. 3c. Compared with Fig. 3a, the spectral shape and peak position remain nearly unchanged, which implies that the Al_2_O_3_ layer and organic adhesive layer can effectively prevent hot electrons that decay from LSPs or SPPs from injecting into monolayer WSe_2_: if hot electron injection has a nonnegligible effect on the considerable upconversion enhancement, the plentiful injected electrons in the WSe_2_ monolayer tend to form trions by capturing neutral excitons, thereby dramatically changing the spectral shape. However, the relationship between the upconversion intensity and excitation power varies significantly; that is, the upconverted emission gradually increases to saturation, and the enhancement accordingly reduces with increasing excitation power, as shown in Fig. 3d. The saturation phenomenon in the upconversion process could be ascribed to the saturated absorption and the exciton radiative lifetime (see Supplementary Fig. S5 for more details).

In addition, the inset of Fig. 3c shows standard PL maps of the plasmonic nanocavity effect on exciton emission excited by the photon energy of 3.04 eV. The nanocubes and monolayer WSe_2_ are marked by red solid lines and white dashed lines, respectively. The spatial resolution is determined by the focal spot size, laser stability, location accuracy, and instrument response function of the microscopy imaging system. In addition, owing to the long-distance diffusion of excitons, the PL signals can be observably enhanced by the Purcell effect when the neighboring regions of the plasmonic nanocavities are excited. Thus, the PL map does not show the desired spatial localization. However, the monolayer WSe_2_ in the nanocavities still exhibits a brighter luminescence, while weaker emission is found in the absence of nanocubes. Very noteworthy here is that the PL signals are enhanced only by the resonance between the emitted photon energy and cavity mode at 1.67 eV. Therefore, the enhancement factor of the PL map by the nanocavities is much smaller than that of the upconverted emission process, which is doubly resonant with plasmonic cavity modes.

In light of the fact that only the regions close to the nanocubes rather than the total excitation region are enhanced, the real upconversion enhancement in the plasmonic cavities should be much larger than the value presented in Fig. 3d. Commonly, to quantify the actual upconversion enhancement in plasmonic cavities, the PL enhancement factor can be defined as^40^

1 $$\left\langle {EF} \right\rangle = \frac{{I_{PC} - I_0}}{{I_0}}\frac{{S_0}}{{S_{PC}}}$$

where *I*_*PC*_ is the total upconversion intensity of monolayer WSe_2_ in the plasmonic cavity, *I*_0_ is the upconversion intensity of the monolayer WSe_2_, *S*_0_ defines the excitation area in our measurements (1.84 μm^2^), and *S*_*PC*_ represents the hotspot area in the plasmonic cavity that enhances the upconversion of the monolayer WSe_2_. For brevity, we assume that *S*_*PC*_ is the area in which the monolayer WSe_2_ contacts the plasmonic cavity (0.0289 μm^2^). An enhancement range of 400~1100-fold can be achieved, corresponding to Fig. 3d.

In addition, the dependence of the upconversion intensity on the excitation power for plasmonic upconverter devices in Fig. 3d can be fitted well by (see Supplementary Fig. S5 for more details)

2 $$I = I_{sat}\frac{f}{{f + f_{sat}}}$$

where *I* and *I*_*sat*_ are the total upconverted emission intensity and the corresponding saturation value, respectively, and *f* and *f*_*sat*_ are the excitation power and the corresponding saturation value, respectively. The optimal fitting parameters of the red dotted curve are *I*_*sat*_ = 3.3 × 10^7^ counts and *f*_*sat*_ = 2.56 mJ/cm^2^. For the excitation power *f* << *f*_*sat*_, Eq. (2) can be simplified to *I* = *I*_*sat*_*f/f*_*sat*_. Based on the fitted slope of the blue dotted line in Fig. 3d, the saturated excitation power can be estimated to be 14.41 mJ/cm^2^, assuming *I*_*sat*_ = 1.4 × 10^7^ counts for monolayer WSe_2_ on SiO_2_/Si (Supplementary Fig. S6). Actually, the measured power-dependent emission intensity (red points) collects the upconverted emission in both the plasmonic cavity and neighboring free space. The real saturated excitation power in our designed doubly resonant plasmonic cavity can be estimated to be 32.9 μJ/cm^2^ (see Supplementary Fig. S7 for more details), which is 2~3 orders of magnitude lower than that of free space.

### Mechanism of enhanced excitonic upconverted emission in plasmonic cavity

**Fig. 4:**
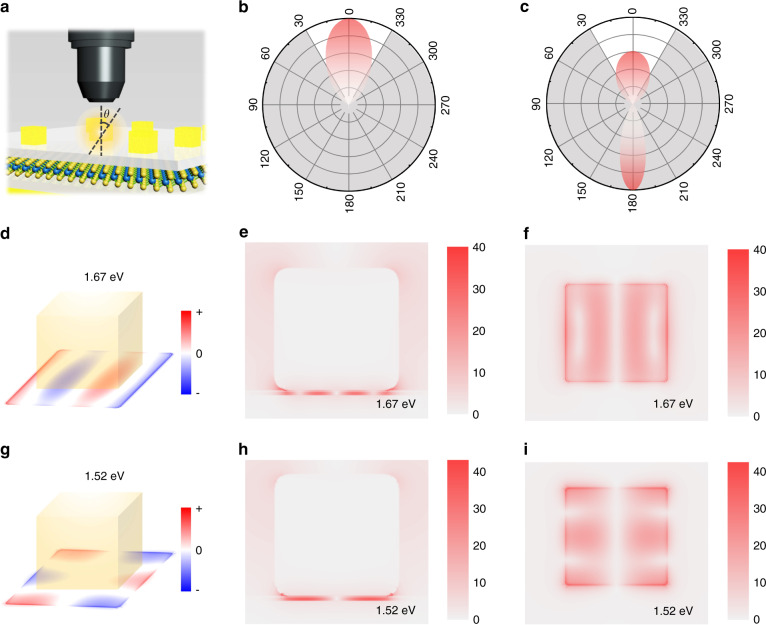
Mechanism of the enhanced upconversion of the plasmonic cavity. **a** Schematic of the setup for collecting upconverted emissions in our experiments. **b**, **c** Far-field angular radiation patterns for monolayer WSe_2_ in the plasmonic cavity (**b**) and free space (**c**). **d**–**f** Charge and field distribution (|E/E_0_|) around the plasmonic cavity for the mode at the emitted photon energy (1.67 eV). **g**–**i** Charge and field distribution (|E/E_0_|) around the plasmonic cavity for the mode at the excitation photon energy (1.52 eV)

In the doubly resonant plasmonic cavity, the upconverted emission enhancement originates from three processes: the enhanced light collection, enhanced excitation rate, and quantum efficiency enhancement arising from the Purcell effect, resulting in an average upconversion enhancement factor of 400~1100-fold. First, as a nanoscale patch antenna, the plasmonic cavity can improve the directionality of emission and thereby enhance light collection for an optical system with a fixed numerical aperture (NA) (Fig. 4a). The radiation pattern of the antenna can be simulated by 3D-finite-difference time-domain (FDTD) simulations (see Supplementary Figs. S8 and S9 for more details). As shown in Fig. 4b, for the in-plane dipole source with a center wavelength of 750 nm and spectral width of 25 nm (similar to the PL spectrum of monolayer WSe_2_), the far-field radiation pattern has a single lobe oriented in the surface-normal direction. The fraction of emitted light collected by the objective lens with NA = 0.5 used in our measurements can be calculated to be 44.4%, which is ~1.7-fold higher than that of monolayer WSe_2_ on Si/SiO_2_.

In addition, to clarify how the plasmonic cavity observably molds the light field and enhances upconverted emission, 3D-FDTD simulations were performed to simulate the charge and field distribution around the plasmonic cavity. In the plasmonic cavity, AuNCs interact with the Au film, and then the image dipoles form and couple with the SPPs of AuNCs, bringing about strong field enhancement with both in-plane and out-plane components (see Supplementary Fig. S10 for more details). Figure 4d, g shows the charge distributions at the monolayer WSe_2_, corresponding to resonant energies of 1.67 and 1.52 eV in the far-field scattering spectrum (Fig. 1f), respectively. The results reveal that the resonance at 1.67 eV can be ascribed to mixed dipolar modes, whereas the resonance at 1.52 eV arises from the coupled quadripolar mode. Figure 4e, h shows the field distribution at the *xz* plane of two plasmonic cavity modes, and Fig. 4f, i presents the field distribution (|E/E_0_|) at monolayer WSe_2_ (see Supplementary Figs. S11 and S12 for more details). These field distributions demonstrate that the plasmonic modes at 1.67 and 1.52 eV confine the excitation light field in the gap with a maximum field enhancement (|E/E_0_|) up to 40. The maximum enhancement of the light intensity (|E/E_0_|^2^) >1600-fold for the mode at excitation photon energy (1.52 eV) can provide a reasonable explanation for the 2~3-order decrease in the saturated excitation power in the plasmonic cavity.

In addition, field enhancements up to 40-fold for the modes at the PL energy (1.52 eV) of the A exciton can yield an appreciable Purcell effect. The quantum efficiency from exciton to upconverted emission can be enhanced by the Purcell effect, which can boost the rate of spontaneous emission by manipulating the local density of optical states according to Fermi’s golden rule. In the resonant cavity, the local density of states can be greatly increased, and the enhancement of the spontaneous emission rate can be expressed as the Purcell factor^53^:

3 $$F = \frac{{\gamma _{{{{\mathrm{cav}}}}}}}{{\gamma _0}} = \frac{3}{{4\pi ^2}}\frac{Q}{{V_{{{{\mathrm{mode}}}}}}}\left( {\frac{\lambda }{n}} \right)^3$$

where *γ*_cav_ and *γ*_0_ are the spontaneous emission rate of the emitter in cavity and free space, respectively, *Q* is the cavity quality factor, *V*_mode_ is the mode volume, *λ* is the resonant wavelength, and *n* is the refractive index of the medium. Without calculating the exact Purcell factor, Eq. (3) illustrates that the plasmonic nanocavity can possess a large Purcell factor owing to the nanoscopic mode volume for a modest cavity quality factor. Thus, 2D excitons in an elaborate plasmonic cavity can exhibit enormous enhancement of the spontaneous emission rate and luminescence.

### Purcell effect modulated exciton relaxation in plasmonic cavity

To gain more insight into the Purcell-enhanced upconverted emission, the exciton relaxation dynamics in the plasmonic cavity and free space were measured by femtosecond pump-probe spectroscopy. In the measurements, to explore the relaxation dynamics of the A excitons in monolayer WSe_2_, the photon energies of pump and probe pulses were chosen to be 3.04 and 1.65 eV, and the pump and probe energy densities were 12.9 and 1.2 μJ/cm^2^, respectively.

**Fig. 5:**
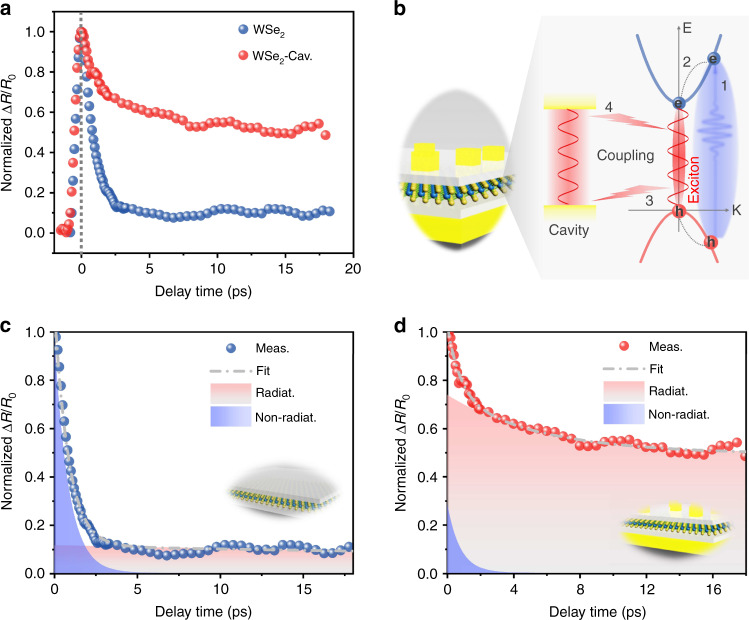
Purcell effect modulated exciton relaxation in the plasmonic cavity. **a** Normalized differential reflection signal Δ*R*/*R*_0_ for monolayer WSe_2_ in free space and the plasmonic cavity. **b** Schematic diagram of the Purcell effect modulated exciton relaxation in the plasmonic cavity: 1. Nonresonant excitation, 2. intraband relaxation of energetic carriers, and 3–4. coupling between the cavity and exciton emission. **c**, **d** Fittings of the exciton relaxation in free space (**c**) and the plasmonic cavity (**d**). The regions filled with gradient red and blue colors represent the radiative and nonradiative contributions, respectively

Figure 5a shows the normalized differential reflection signal Δ*R*/*R*_0_ for monolayer WSe_2_ on SiO_2_/Si (blue points) and monolayer WSe_2_ in the plasmonic cavity (red points). Clearly, the exciton relaxation process in the plasmonic cavity changes dramatically due to the Purcell effect arising from the resonance between the A exciton peak and cavity mode. The schematic diagram of the Purcell effect modulated exciton relaxation in the plasmonic cavity is sketched in Fig. 5b. The energetic electrons and holes are excited by the pump pulse (1) and then relax to excitons (2). These high-density excitons either recombine radiatively with the emission of photons (3) or dissipate non-radiatively through many-body scattering, including multi-exciton annihilation and exciton-exciton annihilation^54,55^. In the resonant plasmonic cavity, the coupling between the cavity and exciton emission (3 and 4) can prominently improve the spontaneous emission rate of excitons through the Purcell effect, thereby reconstructing the competitive relationship between radiative recombination and nonradiative annihilation during the relaxation of excitons.

Generally, considering multi-exciton annihilation (~*N*^*x*^, *x* > 2), exciton-exciton annihilation (~*N*^2^), and exciton radiative recombination (~*N*), the rate equation that describes the exciton relaxation dynamics can be written as follows:

4 $$\frac{{\partial N}}{{\partial t}} = - \alpha N^4 - \beta N^2 - \gamma N$$

where *N* is the exciton density, *α* and *β* are the biexciton-biexciton annihilation rate and exciton-exciton annihilation rate, respectively, and *γ* is the exciton decay rate of single exciton radiative recombination. Compared with radiative recombination, many-body scattering (~*N*^*x*^, *x* ≥ 2) is a much faster process. Therefore, to quantify the time constants of fast nonradiative annihilation and slow radiative recombination, the transient decay dynamics can be approximately fitted by two exponential decay components.

Figure 5c, d shows the fitted exciton relaxations of monolayer WSe_2_ in free space and the plasmonic cavity. Monolayer WSe_2_ in free space (Fig. 5c) undergoes exciton relaxation with a fast decay time constant of 0.86 ps and a slow decay time constant of 87 ps, while the fast and slow decay time constants are 0.78 and 5.7 ps for monolayer WSe_2_ in the plasmonic cavity (Fig. 5d), respectively (see Supplementary Fig. S13 for monolayer WSe_2_ on the Au film). Note that the radiative lifetime of 87 ps of the WSe_2_ monolayer at room temperature is much longer than the intrinsic exciton radiative time (on the order of 1 ps) at cryogenic temperatures. This can be attributed to the thermalized Boltzmann distribution of excitons owing to the exciton-phonon interactions^56–58^. The reduced radiative lifetime indicates a 15-fold radiative decay rate enhancement, and the actual enhancement can be much greater considering that the collected signals comprise excitons coupled to the plasmonic cavity and neighboring excitons without coupling. Accordingly, the constituents of radiative recombination (filled red) and nonradiative annihilation (filled blue) in exciton relaxation are significantly altered; that is, nonradiative annihilation is suppressed and radiative recombination is greatly enhanced in the plasmonic cavity, which agrees with the enhancement of the upconverted emission in Fig. 3.

## Discussion

In summary, the multiphoton upconverted emission of 2D excitons can be greatly enhanced by the elaborate doubly resonant plasmonic nanocavity. An upconverted emission amplification of >1000-fold and a decrease of nearly three orders of magnitude for the saturated excitation power are achieved. This can be attributed to the enhanced light collection, enhanced excitation rate, and quantum efficiency enhancement arising from the Purcell effect. These findings pave the way for the development of excitonic upconversion lasing, nanoscopic thermometry, and sensing and reveal the possibility of optical refrigeration in future 2D electronic- or excitonic-based devices.

## Materials and methods

### Sample preparations

First, a 50 nm Au film and a 5 nm Al_2_O_3_ isolation layer were deposited on the surface of silicon wafers with 300 nm SiO_2_ by electron beam evaporation, the 5 nm Al_2_O_3_ spacer layer is fully oxidized from Al deposited by electron beam evaporation (DE400), the Al thickness is precisely controlled and confirmed by a crystal oscillator sensor (SQS-242) attached to the DE400 system, the thickness of 5 nm Al2O3 is automatically converted by the system with an accuracy <0.1 nm. And then, an exfoliated WSe_2_ monolayer was transferred onto the substrate by a polydimethylsiloxane-assisted drying method. In the following process, AuNCs with a size of 170 nm were uniformly distributed on the surface of the sample by a chemical-assisted method. In a typical process, 58.6 mg NaCl and 45.1 mg PAH were dissolved in 1 ml deionized (DI) water to obtain PAH solutions, 58.6 mg NaCl, and 212.3 mg PSS were dissolved in 1 ml DI water to obtain PSS solutions. Then, the substrate containing the sample was then fully immersed in PAH and PSS solutions alternately, at room temperature, and held for 5 min, after each immersion, the substrate was carefully cleaned with DI water. In this step, an organic adhesive layer was deposited on the substrate surface by a layer-by-layer assembly process^59–62^. The layer thickness was estimated to be on the order of 1 nm^59,62^ (see Supplementary Fig. S2 for more details). Finally, a pipette gun was used to drop the 50 mg/l AuNC DI water solutions onto the substrate at an amount of approximately 100 microliters per square centimeter. Then, the substrate was steamed to dry the solvent on a hot plate at 90 °C in air.

### Dark-field scattering measurements

The dark-field scattering mapping and spectra were measured with a commercial hyperspectral imaging system (Cytoviva, HISV3). The white light was focused by a 100× objective with a high NA (Olympus, MPlanFLN, NA = 0.9). The mapping of the scattering signal was realized with a precise motorized translation stage, and spectral profiles of all pixels could be obtained. Scattering signals were recorded by a spectrometer (Horiba, iHR550) cooled to −60 °C. Scattering spectra of samples were corrected with the substrate using build-in software (Cytoviva, ENVI 4.8).

### Confocal PL maps measurements

PL maps were measured by an ISS Q2 confocal laser scanning system coupled to a Nikon TE2000 microscope with a 60×/1.2 NA WI objective lens. The excitation wavelength was 405 nm (5000 Hz repetition rate), and PL emission signals were collected through a 480 nm longpass edge filter.

### Mirco-PL spectra measurements

The temperature was controlled by a Heating and Freezing Microscope Stage (LINKAM THMS600) system, where the adjustable temperature range is 80–970 K, with an accuracy of 0.1 K. The femtosecond pulses (817 nm, 73 fs, 80 MHz) emitted from a mode-locked oscillator (Tsunami 3941C-25XP) were focused by an infinity-corrected long work distance micro-objective (Mitutoyo, 100×, NA = 0.5) to excite the sample, which was placed in the Heating and Freezing Microscope Stage. The excitation sites were confirmed with an EMCCD camera (Andor Ixon 888) with a micro-objective and matched widefield tube lens (Thorlabs TTL200-A). For spectral measurements, an 800 nm short-pass edge filter (Thorlabs FELH0650) was used to block the excitation light. The filtered light was coupled to a spectrometer (Acton SP2500) equipped with a liquid nitrogen-cooled CCD.

### Ultrafast measurements

Ultrafast pump-probe measurements in reflection configuration were carried out. The femtosecond pulses (817 nm, 73 fs, 80 MHz) were split into two parts. One of them passed through a BBO crystal to produce the 408 nm pump pulses, while the other one was focused on a photonic crystal fiber (Newport SCG-800) to generate the super-continuum white light. The probe pulses were then selected with a 750 ± 10 nm (Thorlabs FB750-10) bandpass filters. The spot size of the focused probe and pump laser was <1 μm. The delay time between pump and probe pulses was controlled by a steeper linear stage (Newport M-ILS150PP). To improve the signal-to-noise ratio, the reflected probe pulses passed through a 650 nm longpass edge filter (Thorlabs FEL0650) and then were detected by a high-sensitivity photomultiplier (Thorlabs PMM02) connected with the phase lock-in amplifier (Stanford SR830).

### Finite-difference time-domain (FDTD) simulations

3D-FDTD simulations were employed to simulate electromagnetic field properties. In the simulation of scattering spectra, reflectance spectra, charge, and electromagnetic field distributions, a total-field scattered-field source with wavelengths ranging from 500 to 900 nm was used. In addition, in-plane dipole source arrays with a center wavelength of 750 nm and spectral width of 25 nm were used to simulate the far-field angular radiation patterns. A perfectly matched layer was set as the boundary conditions, and the mesh size around the plasmonic cavity was 1 nm. The relative permittivities of Si, SiO_2_, Al_2_O_3_, Au, and WSe_2_ monolayers were taken from the literature^63–65^, and the thickness of the WSe_2_ layer was set as 1 nm.

## Supplementary information

Supplementary Information for Giant excitonic upconverted emission from two-dimensional semiconductor in doubly resonant plasmonic nanocavity
