# Supplementary material for: Giant excitonic upconverted emission from two-dimensional semiconductor in doubly resonant plasmonic nanocavity
Source: Light Sci Appl. 2022 Jun 10;11:176. doi: 10.1038/s41377-022-00860-2 (PMC9187628; doi:10.1038/s41377-022-00860-2)
Supplement: Supplementary file 1 — Supplementary Information for Giant excitonic upconverted emission from two-dimensional semiconductor in doubly resonant plasmonic nanocavity [file 41377_2022_860_MOESM1_ESM.pdf]

**Supplementary Information for**

# Giant excitonic upconverted emission from two-dimensional semiconductor in doubly resonant plasmonic nanocavity

Pengfei Qi,[1,2,#] Yuchen Dai,[1,#] Yang Luo,[1,#] Guangyi Tao,[1,3] Liheng Zheng,[1] Donglin Liu,[1] Tianhao Zhang,[3] Jiadong Zhou,[4] Bo Shen,[1] Feng Lin,[1] Zheng Liu,[4] and Zheyu Fang[1,*]

[1] School of Physics, State Key Laboratory for Mesoscopic Physics, Academy for Advanced Interdisciplinary Studies, Collaborative Innovation Center of Quantum Matter, Nano-optoelectronics Frontier Center of Ministry of Education, Peking University, Beijing 100871, China

[2] Institute of Modern Optics, Nankai University, Tianjin Key Laboratory of Micro-scale Optical Information Science and Technology, Tianjin 300350, China

[3] Photonics Research Center, School of Physics, MOE Key Lab of Weak-Light Nonlinear Photonics, and Tianjin Key Lab of Photonics Materials and Technology for Information Science, Nankai University, Tianjin 300071, China

[4] School of Electrical and Electronic Engineering, Nanyang Technological University, Singapore 639798, Singapore

[#] These authors contributed equally: Pengfei Qi, Yuchen Dai and Yang Luo

* Email: zhyfang@pku.edu.cn

# CONTENTS


**S1. SEM images of Au nanocubes and plasmonic cavities ........................................ 1**

**S2. Characterization of the organic adhesive layer ................................................. 2**

**S3. Raman spectra of monolayer $WSe_2$ in free space and plasmonic cavity .......... 4**

**S4. Temperature-dependent upconverted emission spectra ...................................... 5**

**S5. Physical mechanism of saturated upconverted emission .................................... 7**

**S6. Power-dependent PL spectra of monolayer $WSe_2$ ............................................. 9**

**S7. Estimation of real saturated excitation power .................................................. 10**

**S8. Schematics for simulating far-field radiation pattern ..................................... 12**

**S9. Far-field radiation for monolayer $WSe_2$ in free space and plasmonic cavity 13**

**S10. Schematic diagram of AuNCs-substrate interaction ........................................ 14**

**S11. Charge and field distribution around plasmonic cavity at 1.98 eV ............. 15**

**S12. Magnetic field distributions around plasmonic cavity .................................... 16**

**S13. Exciton relaxation for the monolayer $WSe_2$ on Au film ................................ 17**

**S14. Temperature-dependent upconversion at higher excitation photon energy 18**

**S15. Plasmonic-nanocavity-enhanced upconverted emission in other samples ... 19**

**References ................................................................................................... 20**

## S1. SEM images of Au nanocubes and plasmonic cavities

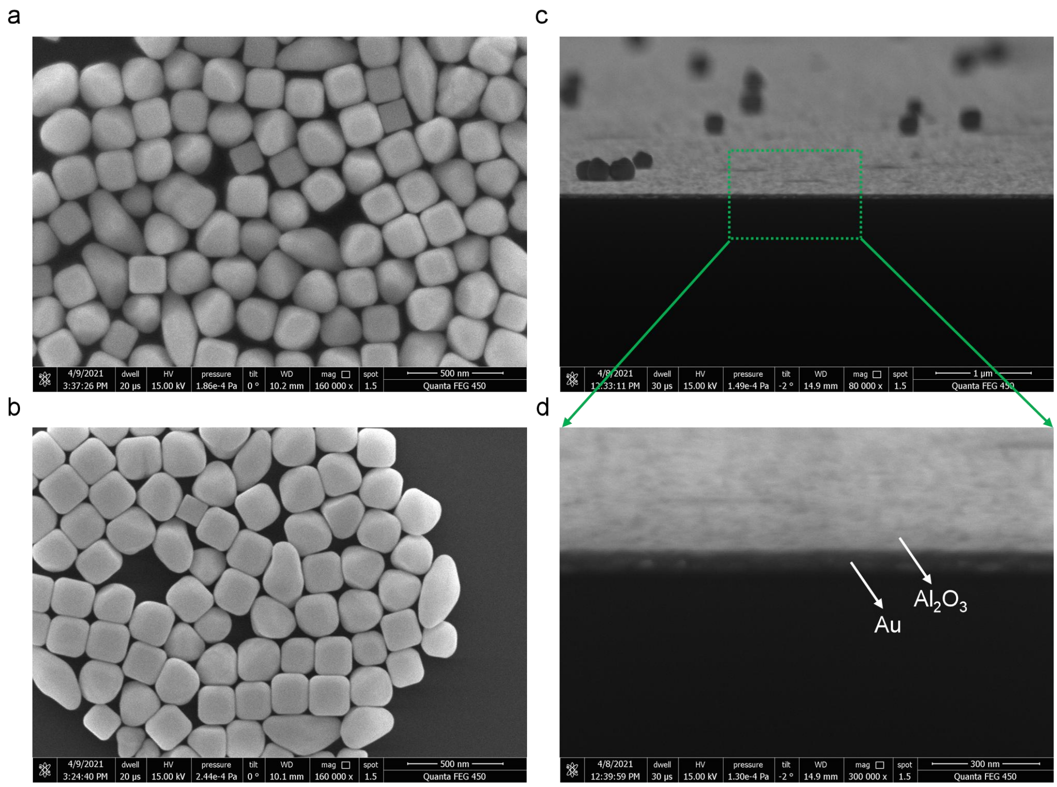


Figure S1. SEM images of Au nanocubes and plasmonic cavities. (a), (b) Top-view SEM images of |the seed-mediated synthetic Au nanocubes. (c), (d) Cross-sectional SEM view of a typical Au nanocube/$Al_2O_3$/Au film plasmonic cavities heterostructure, imaged at a 2° tilted angle. The dark area corresponds to the substrate of Si/$SiO_2$.

## S2. Characterization of the organic adhesive layer

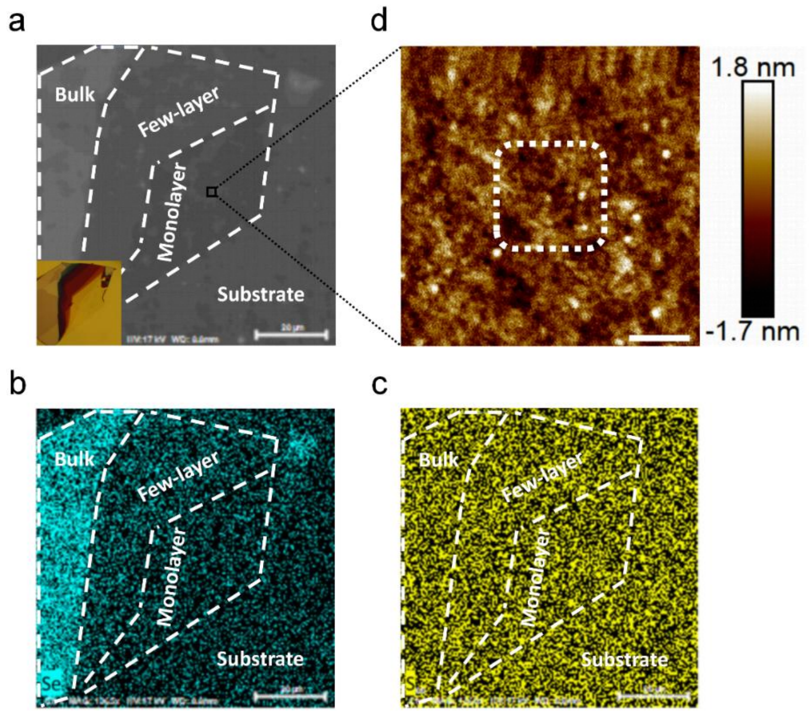


Figure S2. Characterization of the organic adhesive layer. (a-c) EDS analysis results, acceleration voltage 17 kV, scale bar 20 μm, obtained by using a ZEISS MERLIN Compact Field-emission scanning electron microscope and a Bruker SEM QUANTAX EDS probe. (a) SEM image of the scanning area, inset: Optical micrograph. EDS imaging of (b) selenium and (c) sulfur, the atomic ratio of Se to S is 1:1.3. (d) The AFM height image of the organic adhesive layer on a monolayer $WSe_2$, scale bar 100nm, obtained by using a Bruker Dimension Icon atomic force microscope, the white dotted frame indicates the size of an AuNC.

Energy dispersive X-ray spectroscopy (EDS) and atomic force microscopy analysis (AFM) were used to characterize the organic adhesive layer between $WSe_2$ and AuNCs. In EDS analysis, we scanned the element distribution in the area (80 μm × 80 μm) with a $WSe_2$ sample on the substrate (Figure S2a), and used sulfur in PSS $(C_8H_7NaO_3S)_x$ to indicate the distribution of the organic adhesive layer on the substrate surface. In order to eliminate the influence of other sulfur-containing pollutants, the substrate was not further treated by dripping AuNCs solution after soaking in PAH and PSS solution alternately. The substrate was cleaned in advance,

and the $WSe_2$ was transferred to the substrate by the drying transfer method to prevent inducing impurities. The scanning imaging results of selenium show a clear characteristic X-ray signal (Figure S2b), and the signal intensity is obviously different between the bulk and few-layer position areas of $WSe_2$. From the scanning results of sulfur, it can be seen that sulfur is uniformly distributed on $WSe_2$ monolayer, few-layer, bulk and $Al_2O_3$ substrate (Figure S2c), which implies the uniformity of organic adhesive layer. The signal intensity of sulfur in this region is slightly stronger than that of selenium, and the atomic ratio of Se to S is 1:1.3. Considering that the element distribution density of Se in $WSe_2$ is larger than that of S in organic adhesive layer, there should be a generous amount of organic molecular chains adsorbed on the substrate surface.

In order to further characterize the morphology of the organic adhesive layer, we used AFM to scan the height distribution of a small flat region (500 nm × 500 nm) on the $WSe_2$ monolayer (Figure S2d). It can be seen from the results that there are fine flocs uniformly distributed on the sample surface, which is consistent with the micro-area morphology formed by the entanglement of polymer chains on the surface of PAH/PSS thin films.[1,2] The size of an AuNC is indicated by a white dotted frame in Figure S2d. It can be seen that the size of an AuNC is much larger than the gap between organic molecular chain clusters. Therefore, direct contact between AuNCs and $WSe_2$ is effectively prevented.

**S3. Raman spectra of monolayer $WSe_2$ in free space and plasmonic cavity**

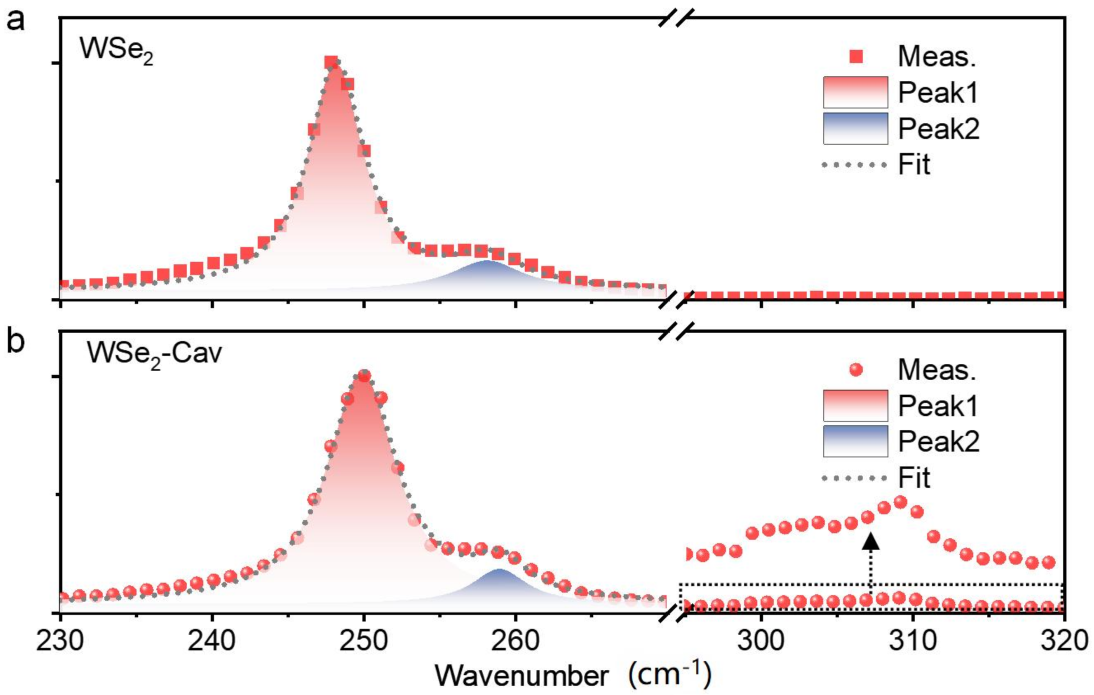


Figure S3. Raman spectra and corresponding Lorenz fitting of the monolayer $WSe_2$ in free space (a) and plasmonic cavity (b). The monolayer $WSe_2$ in free space has a typical Raman spectrum with two dominant peaks at 248.19 $cm^{-1}$ (phonon energy of 30.78 meV) and 258.16 $cm^{-1}$ (phonon energy of 32.01 meV) corresponding to atomic displacements of the in-plane $E^1_{2g}$ and out-of-plane $A_{1g}$ modes (a), respectively. There are slightly blueshifts of 1.71 $cm^{-1}$ and 0.82 $cm^{-1}$ for the monolayer $WSe_2$ plasmonic cavity (b). The peaks located at 303 $cm^{-1}$ and 309 $cm^{-1}$ in (b) can be attributed to the poly(sodium-p-styrenesulfonate) (PSS).

## S4. Temperature-dependent upconverted emission spectra

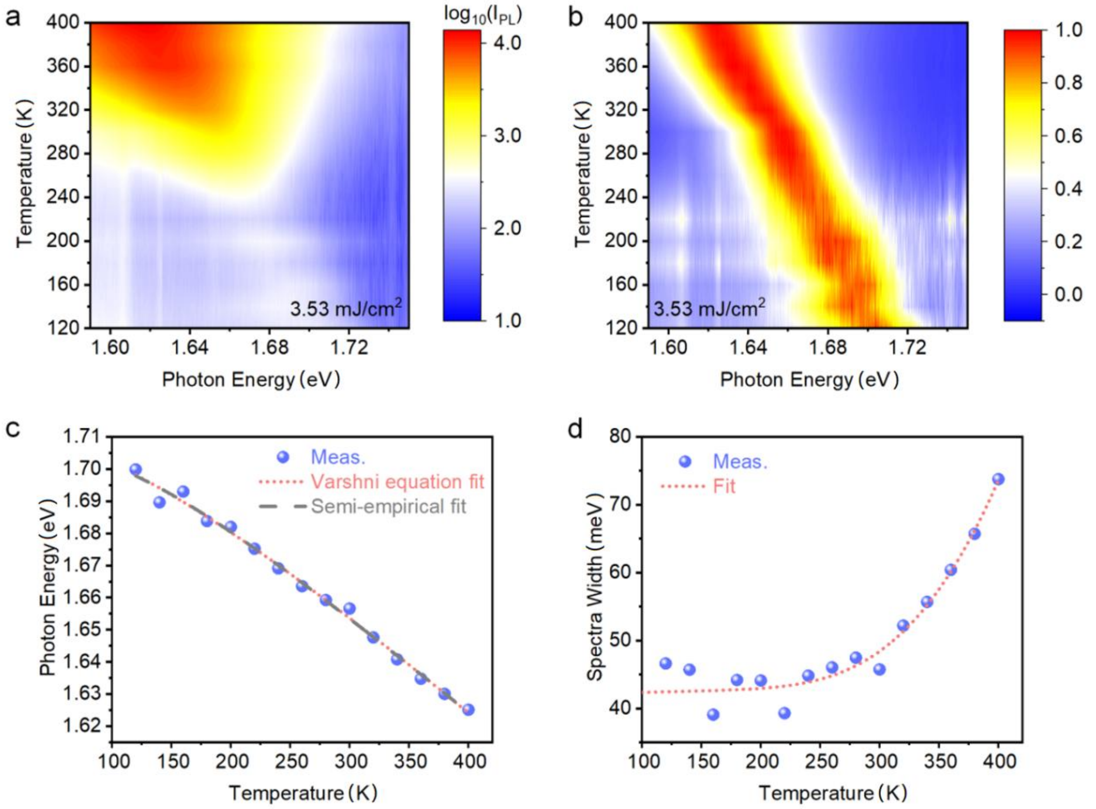


Figure S4. Temperature-dependent upconverted emission spectra. (a) Temperature dependent PL spectra for excitation photon energy at 1.52 eV. (b) Normalized PL spectra of (a). (c), (d) Evolution of peak energy and spectral width with temperature.

The variation of exciton peak can be attributed to temperature-dependent lattice dilatation and electron-phonon interaction. The exciton peak shift (Figure S4c) can be well fitted by the well-known Varshni equation, $E_g(T) = E_g(0) - \alpha T^2/(T+\beta)$, which describes the temperature-dependence of energy gap for various semiconductors.[3] The global optimal parameters of fitting curves are $E_g(0) = 1.712$ eV, $\alpha = 3.57 \times 10^{-4}$ eV/K and $\beta = 249.3$ K. The broadening excitons linewidth results from the interaction of excitons with the longitudinal-acoustical (LA) and longitudinal-optical (LO) phonon modes of lattice for semiconductor without considerable impurity doping and defects. Accordingly, the temperature-dependent

linewidth of excitons can be written as $\Gamma(T) = \Gamma_0 + \gamma_{LA} T + \gamma_{LO} N_{LO}(T)$,[4] where the second term describes the contribution of the interaction between excitons and LA phonons, the third term describes derive from the interaction between excitons and LO phonons with $N_{LO}(T)$ represents the LO phonons occupation with Bose-Einstein distribution, and the constant term arises from scattering due to intrinsic imperfections. As depicted by the red dotted line in Figure S4d, temperature-dependent linewidth can be strictly described by $\Gamma(T)$, where $\Gamma_0$ = 41.92 meV, $\gamma_{LA}$ = 0.04 meV K$^{-1}$, and $\gamma_{LO}$ =5.64 meV.

The semi-empirical fitting function, as an alternative to the Varshni function was introduced to estimate the exciton-phonon coupling strength (Figure S4c).[5,6] The temperature dependence of the bandgap can be described by

$$E_g(T) = E_g(0) - S\langle\hbar\omega\rangle[\coth(\langle\hbar\omega\rangle / 2k_B T) - 1] \quad \text{(S1)}$$

The output exciton-phonon coupling strength $S$ is 1.82, and the average phonon energy is around 29 meV, which is consistent with the Raman measurements in Figure S3, implying the reliability of the estimation.

## S5. Physical mechanism of saturated upconverted emission

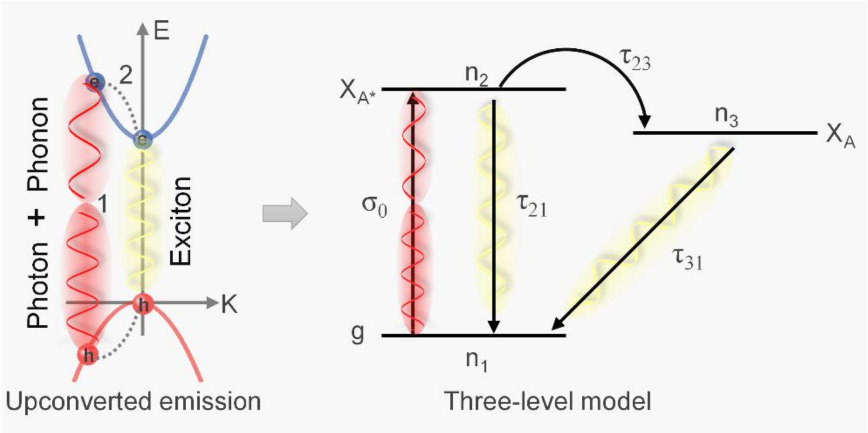


Figure S5. Three-level model for explaining the saturated upconverted emission in monolayer $WSe_2$.

In phonon-assisted excitonic upconverted emission of monolayer $WSe_2$, the electrons at ground state are excited by absorbing a photon and phonons simultaneously and relax as excitons, where the photon energy $\hbar\omega_1$ locates at the long-wavelength tail of the absorption spectrum. Subsequently, the formed excitons can recombine via spontaneous emission of an upconverted photon with energy $\hbar\omega_2 > \hbar\omega_1$. As shown in Figure S5, this physical scenario can be regarded as a three-level model with two excited-state levels $X_{A^*}$, $X_A$ and a mutual ground-state level g, where $X_{A^*}$ is a metastable state.

The saturated upconverted emission can be well explained based on three-level model. Electrons of the monolayer $WSe_2$ on the ground state g simultaneously absorb photons and phonons, and jump to the excited state $X_{A^*}$ with an absorption section $\sigma_0$, and then transit to excitonic state $X_A$ with a large probability of $1/\tau_{23}$. Only a small number of carriers directly relax to ground state. Because of the probability of $1/\tau_{21}$ being very small, a large number of excitons on the state $X_A$ form and accumulate. Under a strong incident light intensity, the excitonic state $X_A$ will reach a saturation value and lead to the saturated absorption and upconverted emission.

The rate equations for describing the change of state density $n_1$, $n_2$ and $n_3$ for the state of g, $X_{A*}$ and $X_A$ can be written as[7,8]

$$\frac{\partial n_2}{\partial t} = \frac{\sigma_0}{\hbar\omega_1} f\left(n_1 - n_2\right) - \frac{n_2}{\tau_{21}} - \frac{n_2}{\tau_{23}} \tag{S2}$$

$$\frac{\partial n_3}{\partial t} = \frac{n_2}{\tau_{23}} - \frac{n_3}{\tau_{31}} \tag{S3}$$

$$N = n_1 + n_2 + n_3 \tag{S4}$$

Where $f$ is the excitation power, and $N$ is the total state density of the system that can be excited.

Considering the $\tau_{31}$ and $\tau_{23}$ is much larger than $\tau_{21}$, the solutions of the above rate equations can be obtained in the steady-state condition ( $\partial/\partial t = 0$ )[7,8]

$$n_1 = \frac{N}{1 + f/f_{sat}} \tag{S5}$$

$$n_2 \approx 0 \tag{S6}$$

$$n_3 = \frac{N}{1 + f_{sat}/f} \tag{S7}$$

Where $f_{sat} = \hbar\omega_1/(\sigma_0 \tau_{31})$ is the saturated excitation power of the three-level system.

Providing the quantum efficiency that excitons can recombine via spontaneous emission is $\eta$, the upconversion intensity can be written as

$$I = \eta n_3 = \frac{\eta N f}{f + f_{sat}} = I_{sat} \frac{f}{f + f_{sat}} \tag{S8}$$

Where $I_{sat} = \eta N$ is the saturated upconverted emission intensity.

## S6. Power-dependent PL spectra of monolayer $WSe_2$

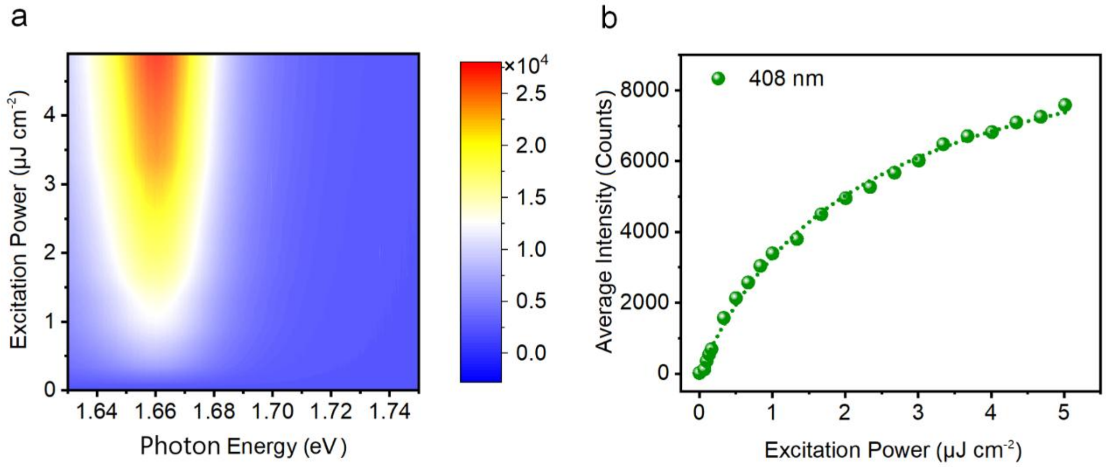


Figure S6. Power-dependent PL spectra of monolayer $WSe_2$ for excitation photon energy of 3.04 eV. (a) Excitation power-dependent unconverted PL spectra for monolayer $WSe_2$. (b) Exitation power-dependent integrated unconverted PL intensity for monolayer $WSe_2$, which can be well fitted by equation (S1). The saturated upconverted emission intensity $I_{sat} = 1.4 \times 10^7$ counts for monolayer $WSe_2$ can be obtained.

## S7. Estimation of real saturated excitation power

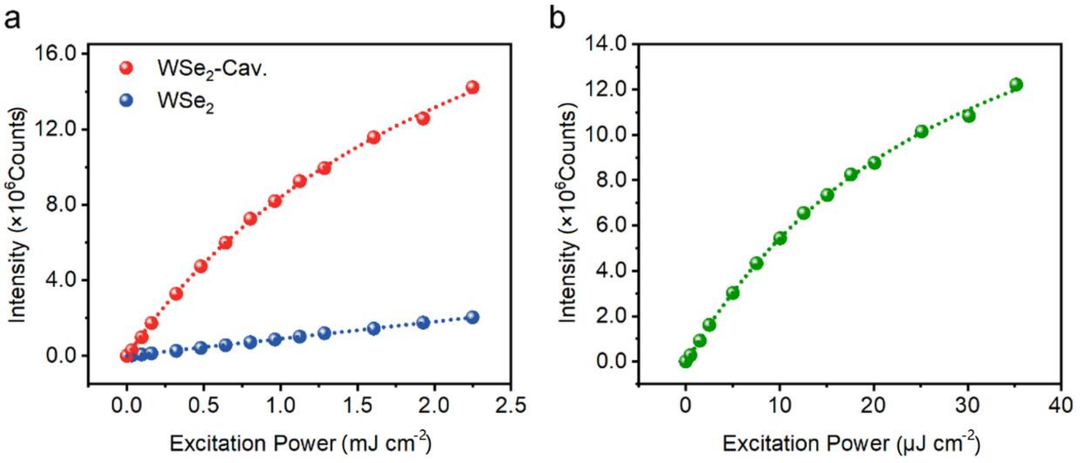


Figure S7. Estimation of real saturated excitation power. (a) Excitation power-dependent integrated upconverted PL intensity for monolayer $WSe_2$ in free space and plasmonic cavity. (b) Actual excitation power-dependent integrated upconverted PL intensity for monolayer $WSe_2$ in the designed plasmonic cavity.

The dependence of upconversion intensity on excitation power for monolayer $WSe_2$ in free space and plasmonic cavity in Figure S7a can be well fitted by equation (S8). In light of the fact that the collected PL spectra in plasmonic cavity (Figure S7a) are comprised of the excitons coupled to plasmonic cavity and neighbouring excitons without coupling, the measured dependence of upconversion intensity on excitation power for plasmonic upconverter devices can be revised as follows

$$I_{meas} = I_{cav} + I_{free} = I_{sat-cav}\frac{pf}{pf + f_{sat-cav}} + I_{sat-free}\frac{(1-p)f}{(1-p)f + f_{sat-cav}} \qquad \text{(S9)}$$

Where $I_{cav}$ and $I_{free}$ are the PL intensities of the excitons coupled to plasmonic cavity and neighbouring excitons without coupling, $I_{sat-cav}$ and $I_{sat-free}$ are the saturated PL intensities for plasmonic cavity and neighbouring excitons without coupling, $f_{sat-cav}$ and $f_{sat-cav}$ are the saturated excitation power for plasmonic cavity and neighbouring excitons without coupling, $p$ is the proportion of the actual

excitation photon energy used to excite the exciton in plasmonic cavity, which can be approximate as $S_{PC}/S_0$, $S_0$ defines the excitation area in our measurements, while $S_{PC}$ represents the hotspot area in plasmonic cavity.

Finally, the actual excitation power-dependent integrated upconverted PL intensity for monolayer $WSe_2$ in plasmonic cavity can be calculated from Figure S7(a), as shown in Figure S7(b). Accordingly, the real saturated excitation power in our designed doubly resonant plasmonic cavity can be estimated as 32.9 μJ $cm^{-2}$, which is reduced by 2 ~ 3 orders of magnitude compared with free space.

## S8. Schematics for simulating far-field radiation pattern

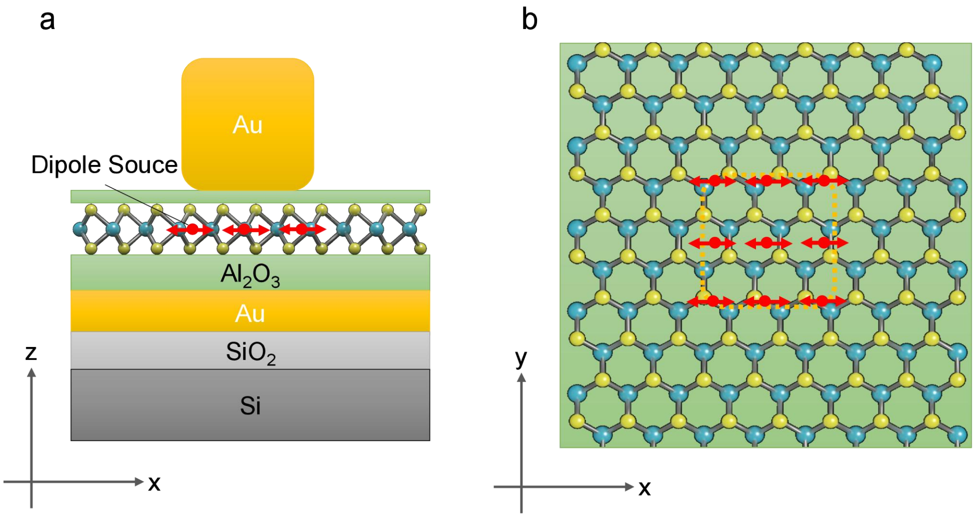


Figure S8. Schematics for simulating far-field radiation pattern. The in-plane dipole source arrays with center wavelength of 750 nm and spectral width of 25 nm were adopted.

## S9. Far-field radiation for monolayer $WSe_2$ in free space and plasmonic cavity

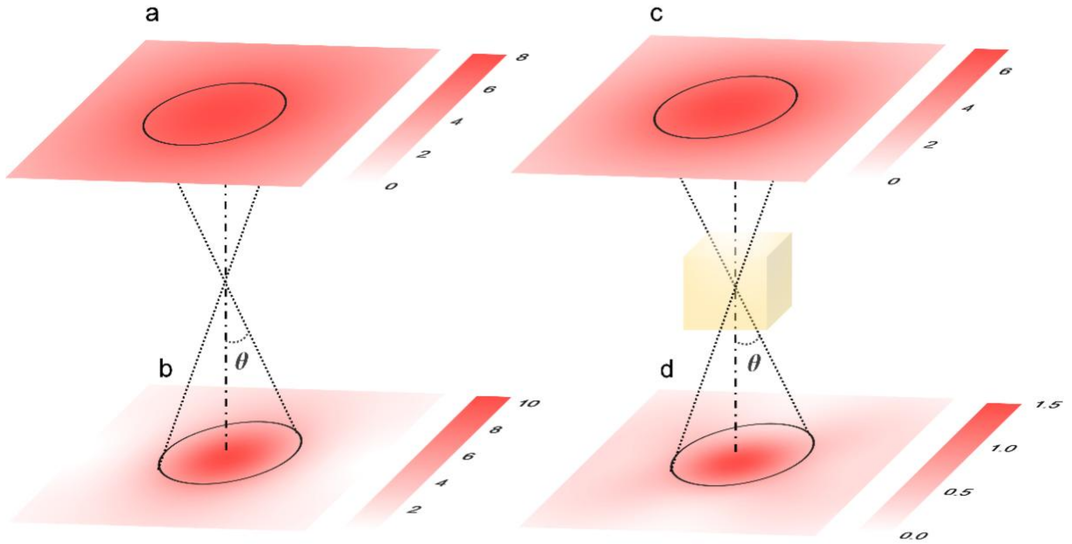


Figure S9. Far-field radiation patterns ($|E/E_0|$) for monolayer $WSe_2$ in free space and plasmonic cavity. (a), (b) Far-field radiation patterns for a monitor placed on the top (a) and bottom (b) of the monolayer $WSe_2$. (c), (d) Far-field radiation patterns for a monitor placed on the top (c) and bottom (d) of the monolayer $WSe_2$ in plasmonic cavity. The average intensity around the angle $\theta$ can be calculated and plotted as the far-field angular radiation patterns in Figures 4b and 4c.

## S10. Schematic diagram of AuNCs-substrate interaction

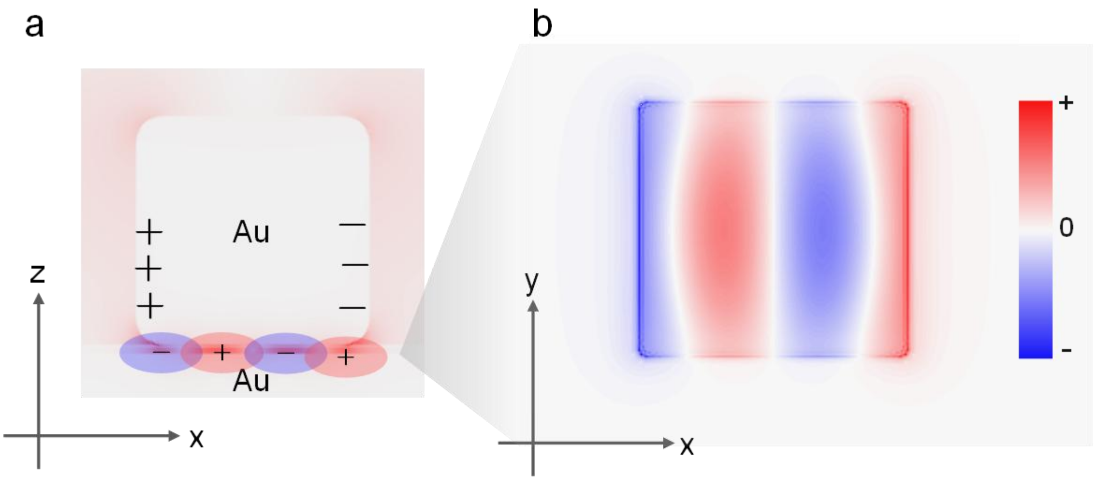


Figure S10. (a) Schematic diagram of AuNCs-substrate interaction for the cavity mode at 1.67 eV. (b) Charge distribution at the monolayer $WSe_2$ for the cavity mode at 1.67 eV.

As shown in Figures S10(a) and S10(b), the image dipole will form in substrate and couple with the SPR of Au nanocubes, leading to strong field enhancement. The fields shown in Figures 4f and 4i have both in-plane and out-plane electric field components. Therefore, the confined electromagnetic field can interact with the 2D exciton with in-plane dipole. It has been demonstrated that the monolayer $MoS_2$ based fluorescent emitter and photodetector can be enhanced by Ag nanoparticles-Au film cavities.[9]

## S11. Charge and field distribution around plasmonic cavity at 1.98 eV

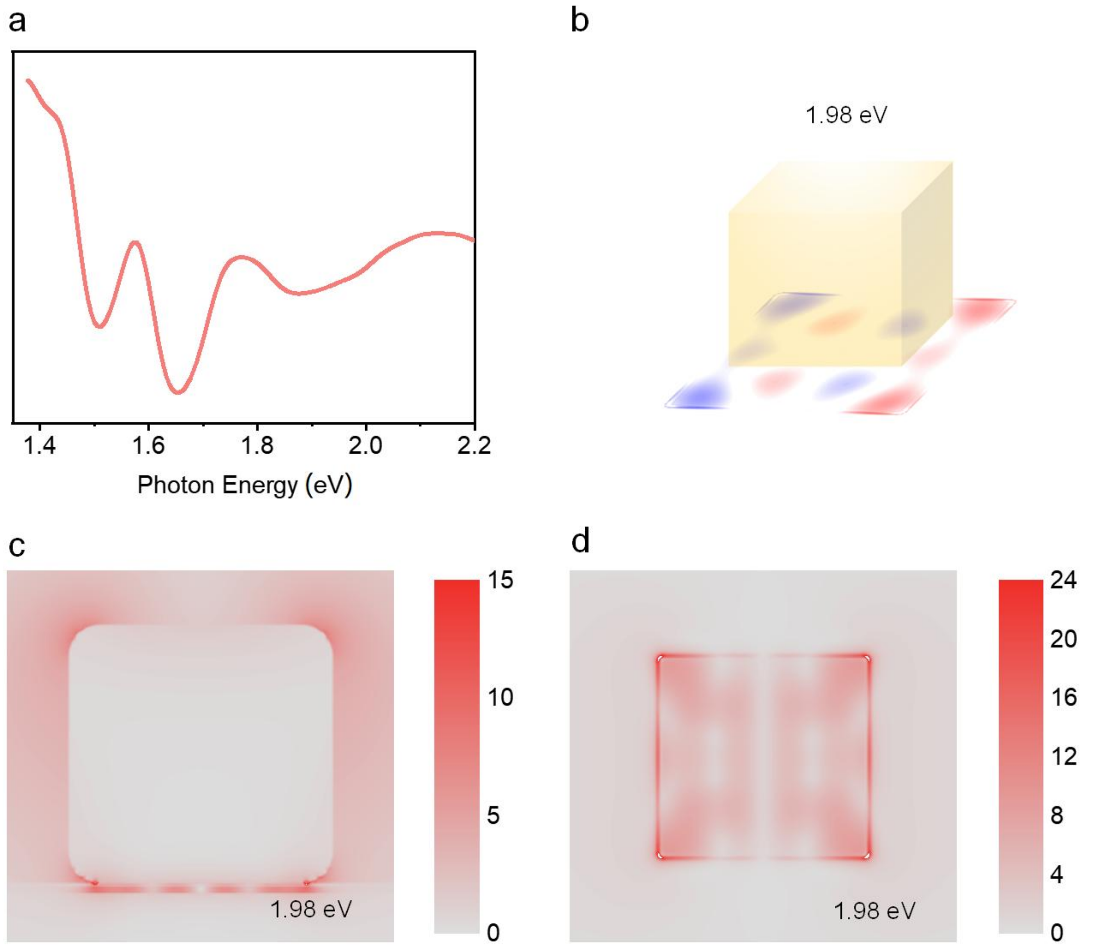


Figure S11. (a) Reflectance spectra of plasmonic upconverter devices simulated by the finite-difference time-domain (FDTD) method. (b), (c), (d) charge and field distribution ($|E/E_0|$) around plasmonic cavity for the mode at 1.98 eV.

## S12. Magnetic field distributions around plasmonic cavity

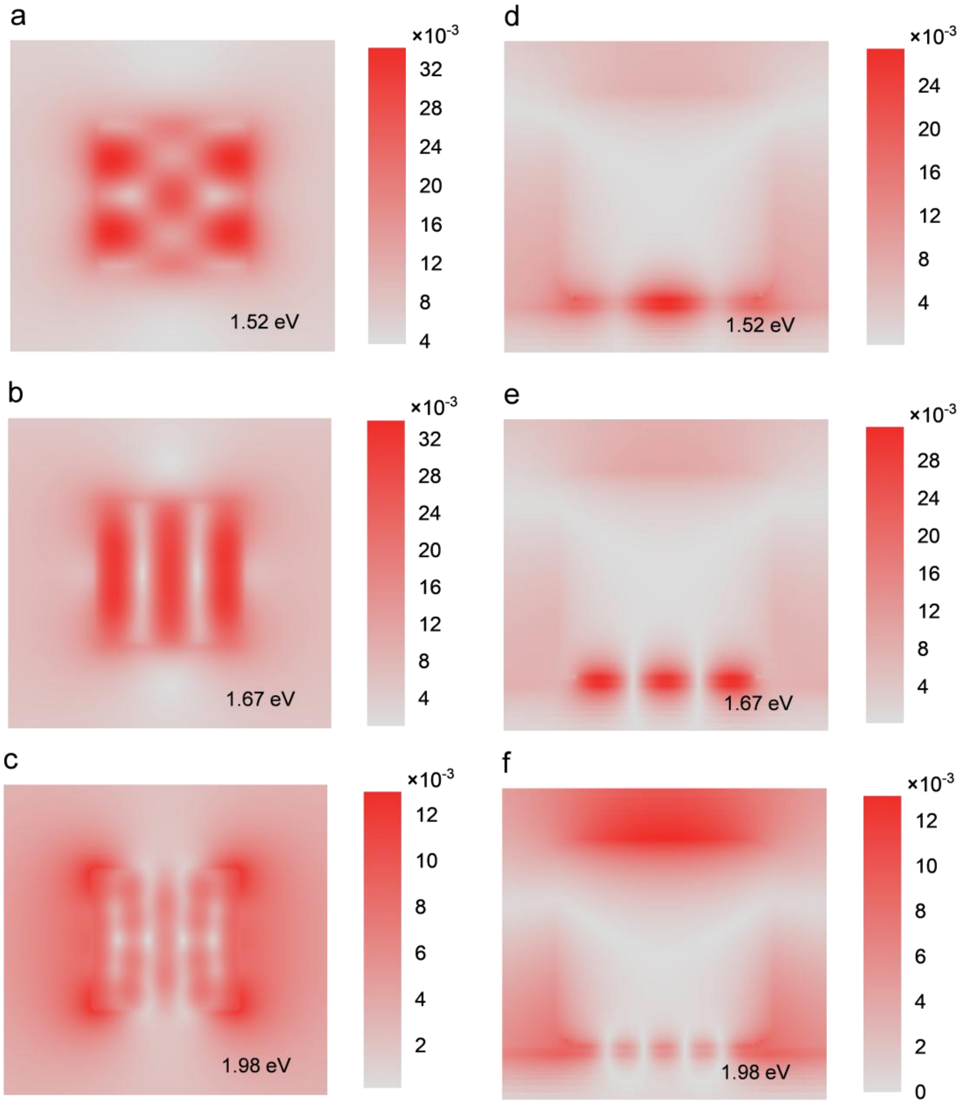


Figure S12. Magnetic field distributions around plasmonic cavity. (a), (d) Magnetic field distributions around plasmonic cavity at 1.52 eV. (b), (e) Magnetic field distributions around plasmonic cavity at 1.67 eV. (c), (f) Magnetic field distributions around plasmonic cavity at 1.98 eV. (a), (b), (c) corresponds to the magnetic field distributions at the monolayer $WSe_2$ plane, and (d), (e), (f) corresponds to the magnetic field distributions at the xz plane.

## S13. Exciton relaxation for the monolayer $WSe_2$ on Au film

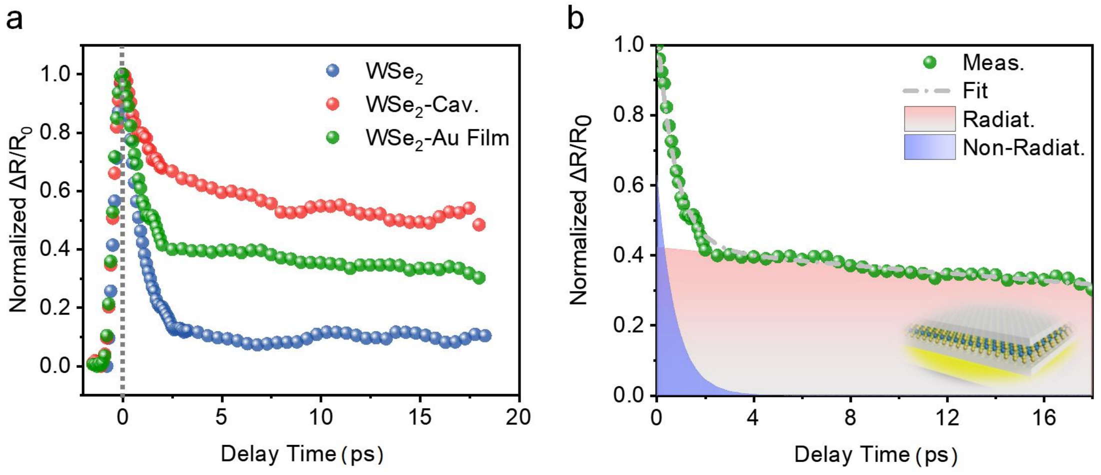


Figure S13. (a) Normalized differential reflection signal $\Delta R/R_0$ for the monolayer $WSe_2$ on Au film (green points). (b) Fittings of the exciton relaxation for the monolayer $WSe_2$ on Au film. The monolayer $WSe_2$ on Au film undergoes the exciton relaxation with a fast decay time constant of 0.76 ps and a slow decay time constant of 62 ps.

## S14. Temperature-dependent upconversion at higher excitation photon energy

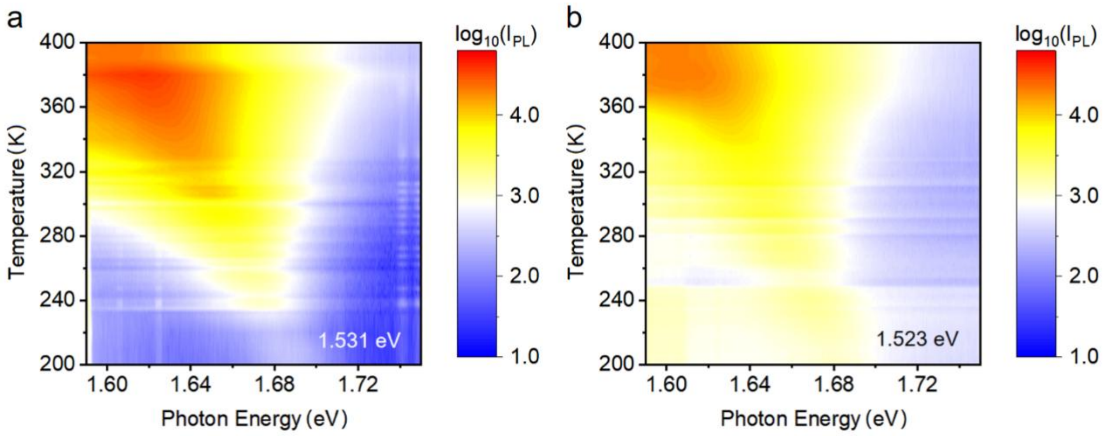


Figure S14. Temperature-dependent upconversion spectra at excitation photon energy of 1.531 eV (a) and 1.523 eV (b).

## S15. Plasmonic-nanocavity-enhanced upconverted emission in other samples

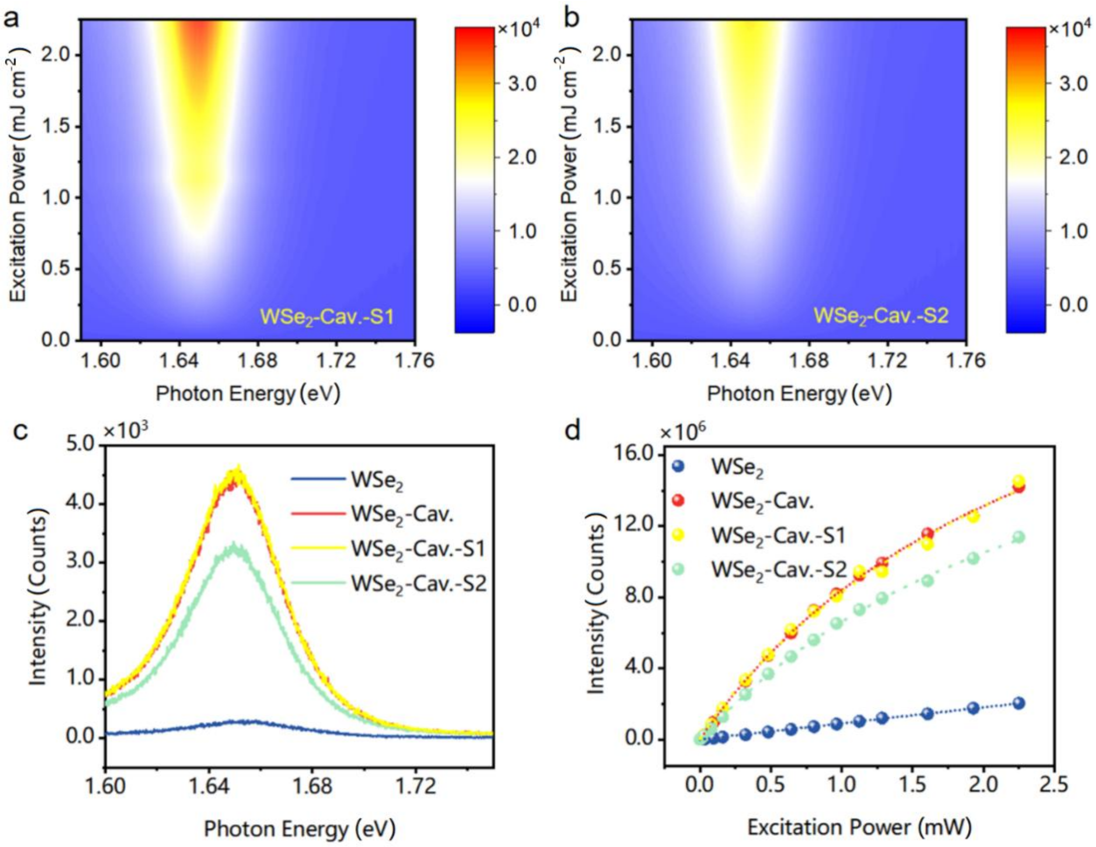


Figure S15. Excitation power-dependent upconverted emission spectra for monolayer $WSe_2$ in the designed plasmonic cavity of supplementary sample S1 (a) and S2 (b). (c) Enhanced upconverted emission spectra of supplementary sample S1 and S2 at the condition of Figure 3b. (d) Excitation power-dependent integrated upconverted emission intensity for supplementary sample S1 and S2.

## References


1 Tristan, F., Menchaca, J. L., Cuisinier, F. & Perez, E. Granular structure of self-assembled PAA/PAH and PSS/PAH nascent films imaged in situ by LC-AFM. *J Phys Chem B* **112**, 6322-6330, doi:10.1021/jp710195e (2008).

2 Lee, J. S. *et al.* Layer-by-layer assembled charge-trap memory devices with adjustable electronic properties. *Nature Nanotechnology* **2**, 790-795, doi:10.1038/nnano.2007.380 (2007).

3 Varshni, Y. P. Temperature dependence of the energy gap in semiconductors. *Physica* **34**, 149-154, doi:https://doi.org/10.1016/0031-8914(67)90062-6 (1967).

4 Rudin, S., Reinecke, T. L. & Segall, B. Temperature-dependent exciton linewidths in semiconductors. *Phys Rev B* **42**, 11218-11231, doi:10.1103/physrevb.42.11218 (1990).

5 Selig, M. *et al.* Excitonic linewidth and coherence lifetime in monolayer transition metal dichalcogenides. *Nature Communications* **7**, 13279, doi:10.1038/ncomms13279 (2016).

6 Helmrich, S. *et al.* Exciton–phonon coupling in mono- and bilayer MoTe2. *2D Materials* **5**, doi:10.1088/2053-1583/aacfb7 (2018).

7 Shen, Y.-R. The principles of nonlinear optics. *New York* (1984).

8 Hu, H. *et al.* Far-field nanoscale infrared spectroscopy of vibrational fingerprints of molecules with graphene plasmons. *Nat Commun* **7**, 12334, doi:10.1038/ncomms12334 (2016).

9 Wu, Z.-Q. *et al.* Gap-Mode Surface-Plasmon-Enhanced Photoluminescence and Photoresponse of MoS2. *Advanced Materials* **30**, 1706527, doi:https://doi.org/10.1002/adma.201706527 (2018)
